# Comparison of linkage disequilibrium estimated from genotypes versus haplotypes for crossbred populations

**DOI:** 10.1186/s12711-022-00703-z

**Published:** 2022-02-08

**Authors:** Setegn Worku Alemu, Piter Bijma, Mario P. L. Calus, Huiming Liu, Rohan L. Fernando, Jack C. M. Dekkers

**Affiliations:** 1 AL Rae Centre for Genetics and Breeding, Massey University, 10 Bisley Drive, Hamilton, 3240 New Zealand; 2 Animal Breeding and Genomics, Wageningen University and Research, 6700 AH Wageningen, The Netherlands; 3 Department of Molecular Biology and Genetics, Aarhus University, 8830 Tjele, Denmark; 4 Department of Animal Science, Iowa State University, Ames, IA 50011 USA

## Abstract

**Background:**

Linkage disequilibrium (LD) is commonly measured based on the squared coefficient of correlation $\left({r}^{2}\right)$ between the alleles at two loci that are carried by haplotypes. LD can also be estimated as the ${r}^{2}$ between unphased genotype dosage at two loci when the allele frequencies and inbreeding coefficients at both loci are identical for the parental lines. Here, we investigated whether ${r}^{2}$ for a crossbred population (F1) can be estimated using genotype data. The parental lines of the crossbred (F1) can be purebred or crossbred.

**Methods:**

We approached this by first showing that inbreeding coefficients for an F1 crossbred population are negative, and typically differ in size between loci. Then, we proved that the expected ${r}^{2}$ computed from unphased genotype data is expected to be identical to the ${r}^{2}$ computed from haplotype data for an F1 crossbred population, regardless of the inbreeding coefficients at the two loci. Finally, we investigated the bias and precision of the ${r}^{2}$ estimated using unphased genotype versus haplotype data in stochastic simulation.

**Results:**

Our findings show that estimates of ${r}^{2}$ based on haplotype and unphased genotype data are both unbiased for different combinations of allele frequencies, sample sizes (900, 1800, and 2700), and levels of LD. In general, for any allele frequency combination and ${r}^{2}$ value scenarios considered, and for both methods to estimate ${r}^{2}$, the precision of the estimates increased, and the bias of the estimates decreased as sample size increased, indicating that both estimators are consistent. For a given scenario, the ${r}^{2}$ estimates using haplotype data were more precise and less biased using haplotype data than using unphased genotype data. As sample size increased, the difference in precision and biasedness between the ${r}^{2}$ estimates using haplotype data and unphased genotype data decreased.

**Conclusions:**

Our theoretical derivations showed that estimates of LD between loci based on unphased genotypes and haplotypes in F1 crossbreds have identical expectations. Based on our simulation results, we conclude that the LD for an F1 crossbred population can be accurately estimated from unphased genotype data. The results also apply for other crosses (F2, F3, Fn, BC1, BC2, and BCn), as long as (selected) individuals from the two parental lines mate randomly.

## Background

Linkage disequilibrium (LD) is the non-random association of alleles at different loci within haplotypes. LD plays an important role in both population and quantitative genetics. In population genetics, LD can for example be used to detect selection [1]. In quantitative genetics, LD has been used to map quantitative trait loci [1–3] and for marker-assisted selection [4] and genomic selection [5]. Thus, knowledge of LD is required for diverse applications in genetics.

LD is traditionally measured based on the comparison of the observed haplotype frequencies with the expected haplotype frequencies under linkage equilibrium. A common statistical measure of LD is the co-variance between loci, $D$, which is equal to the excess of coupling phase haplotypes, ${D}_{ij}={P}_{ij}-{P}_{i}{P}_{j}$, where ${P}_{ij}$ refers to the frequency of gametes (haplotypes) that carry the pair of alleles $i$ and $j$ at the two loci, ${P}_{i}$ and ${P}_{j}$ refer to the frequency at locus $i$ and locus $j$, respectively, and ${P}_{i}{P}_{j}$ is the expected frequency of this haplotype under linkage equilibrium [6]. Another common measure is the squared coefficient of correlation (${r}^{2}$) between the alleles at the two loci within haplotypes, ${r}_{ij}^{2}$= $\frac{{D}_{ij}^{2}}{{P}_{i}\left(1-{P}_{i}\right){P}_{j}\left(1-{P}_{j}\right)}$ [7].

To calculate $D$ and ${r}^{2}$ using the expressions given above, the haplotypes carried by the individuals must be known. However, Rogers and Huff [8] showed that LD can also be estimated by correlating unphased genotype dosages at the two loci, which makes the computation simple and fast. They demonstrated that LD estimated from unphased genotypes yields very similar results to LD estimated from haplotypes. In their derivation, however, they assumed equal inbreeding coefficients for the two loci and equal allele frequencies for the paternal and maternal gametes that created the population. In this context, the inbreeding coefficient measures the departure from Hardy–Weinberg equilibrium and, thus, can take positive or negative values. However, for crossbred individuals inbreeding coefficients can differ between the two loci, and paternal and maternal allele frequencies can differ because the two parents come from different lines.

Here, we investigated whether LD in crossbred populations can be estimated using unphased genotype data. We assumed that sires and dams of the crossbreds originate from two distinct lines but are otherwise mated to each other at random. We address this question in three steps. First, we derive the inbreeding coefficients of crossbreds, showing that they take negative values that typically differ between loci. As a result, the derivation of Rogers and Huff [8] cannot be used to demonstrate the equivalence of genotype-based LD to haplotype-based LD for a crossbred population. Second, we show theoretically that LD computed from genotype frequencies has the same expected value for a given dataset as LD computed from haplotype frequencies, even for a crossbred population. Finally, we investigate the precision and potential bias of LD estimated from unphased genotype data versus haplotype data, using stochastic simulation.

## Methods

### Inbreeding coefficients for a crossbred population

Consider two outbred lines, $A$ and $B$. We want to investigate the inbreeding coefficients for two bi-allelic loci, $M$ and $N$, in the F1 crossbred offspring that result from the crossing of random individuals from two parental lines. With alleles denoted *0* and *1*, ${p}_{AM}$ is the frequency of allele *1* at locus $M$ in line $A$, and ${p}_{BM}$ is the frequency of allele *1* at locus $M$ in line $B$. The expected frequency of allele *1* at locus $M$ in the crossbreds then is ${p}_{M}=\frac{{p}_{AM}+{p}_{BM}}{2}.$ With random mating between individuals from the two parental lines, the frequency of genotype *11* in the crossbred**s** is ${p}_{AM}{p}_{BM}.$ The deviation of this frequency from Hardy–Weinberg equilibrium follows from [6, 9].

$${p}_{AM}{p}_{BM}={p}_{M}^{2}+{p}_{M}\left(1-{p}_{M}\right){f}_{M},$$

where ${f}_{M}$ is the inbreeding coefficient at locus $M$ in the crossbreds.

The inbreeding coefficient follows from solving this expression for ${f}_{M}$, substituting ${p}_{M}=\frac{{p}_{AM}+{p}_{BM}}{2}$, and simplifying the expression, giving:

$${f}_{M}=\frac{-{\left({p}_{AM}-{p}_{BM}\right)}^{2}}{\left({p}_{AM}+{p}_{BM}\right)\left(2-{p}_{AM}+{p}_{BM}\right)}.$$

Similarly, $${f}_{N}=\frac{-{\left({p}_{AN}-{p}_{BN}\right)}^{2}}{\left({p}_{AN}+{p}_{BN}\right)\left(2-{p}_{AN}+{p}_{BN}\right)}.$$

Note that the numerators of ${f}_{M}$ and ${f}_{N}$ are always negative, except when ${p}_{AM}={p}_{BM}$ and ${p}_{AN}={p}_{BN}$, while the denominators are always positive. This shows that the inbreeding coefficients of crossbreds are negative, meaning that heterozygosity is greater than would be expected under Hardy–Weinberg equilibrium (for example ${p}_{AM}=0.05$, ${p}_{BM}=0.09$, ${p}_{AN}=0.25,$ and ${p}_{BN}=0.29$ yields ${f}_{M}=-0.0056$ and ${f}_{N}=-0.0015$).

We investigated under which conditions the inbreeding coefficients at the two loci are equal by solving the expression ${f}_{N}={f}_{M}$ for the allele frequencies, using Wolfram Mathematica (www.wolfram.com). Apart from the trivial solutions of $p$ = 0, $p$ = 1, and equal allele frequencies at both loci, we found only three solutions (see Appendix 1). Hence, this result demonstrates that the inbreeding coefficients at two arbitrary loci in a crossbred population will usually be different. This implies that the derivation of Rogers and Huff [8] cannot be used to demonstrate the equivalence of genotype-based LD to haplotype-based LD for a crossbred population.

### Haplotype-based linkage disequilibrium

In this section, we show that the expected LD based on ${r}^{2}$ computed from the genotype frequencies of the crossbred population is identical to the true ${r}^{2}$ based on haplotype frequencies, even when the inbreeding coefficients differ between the two loci. Note that we consider the true (*i.e.,* population) value of ${r}^{2}$ here, rather than an estimate from a sample. As we consider bi-allelic loci, we have four haplotype frequencies for each line, denoted $r$, $s$, $t$, and $u$ for line $A$, and using ${^{\prime}}$ to refer to frequencies for line $B$, we have haplotype frequencies $r{^{\prime}}$, $s{^{\prime}}$, $t{^{\prime}}$, and $u{^{\prime}}$ for line $B$. Table 1 shows expressions for the marginal frequency for each of the alleles. Although the expressions for the marginal frequencies in Table 1 can be simplified by formulating them in terms of allele frequencies, we stick to the haplotype frequencies to facilitate comparison with results for the genotype-based ${r}^{2}$.

**Table 1:** Haplotype frequencies and marginal allele frequencies for line $A$^a^

Alleles at locus *M*	Alleles at locus *N*		
	0	1	Marginal frequency
0	$r$	$s$	$r+s$
1	$t$	$u$	$t+u$
Marginal frequency	$r+t$	$s+u$	$r+s+t+u=1$

^a^Corresponding symbols for line $B$ are denoted by ${^{\prime}}$

Crossbred genotypes consist of two sets of haplotypes, one from each parental line, which may have a different ${r}^{2}$. By definition, the ${r}^{2}$ in the crossbreds depends on the (co)variances between loci in the crossbred population, so we cannot simply average the ${r}^{2}$ of the two parental lines. From the definitions of correlation, variance, and covariance, it follows that the ${r}^{2}$ for the crossbred population equals the square of the average of the covariances between haplotypes for each of the two lines, divided by the product of the average variance across the two lines at each locus. For line $A$, the covariance between haplotypes (i.e. $D$*)* follows from Table 1 as $u-\left(t+u\right)\left(s+u\right)$, where $u$ is the expectation of the cross product of the allele frequencies at each locus, while $\left(t+u\right)\left(s+u\right)$ is the cross product of the expectations of these allele frequencies (expected haplotype frequency in line $A$ under linkage equilibrum). Hence, this result follows immediately from the definition of a covariance. The covariance ($D$) for line $B$ is analogous, using symbols denoted by ${^{\prime}}$. The variance in allele count follows from the binomial distribution with *n* = 1 for haplotypes and are thus equal to $p\left(1-p\right)$, $p$ denoting the allele frequency. For line $A$ the variance equals $\left(s+u\right)\left(r+t\right)$ for locus $N$, and $\left(t+u\right)\left(r+s\right)$ for locus $M$, with analogous equations for line $B$. Using these values in the haplotype-based ${r}^{2}$ for the crossbred population yields the following true ${r}^{2}$ in the crossbred population:

1 $${r}_{hap}^{2}=\frac{{\left[\left(u-\left(t+u\right)\left(s+u\right)\right)+\left({u}{^{\prime}}-\left({t}{^{\prime}}+{u}{^{\prime}}\right)\left({s}{^{\prime}}+{u}{^{\prime}}\right)\right)\right]}^{2}}{\left[\left(s+u\right)\left(r+t\right)+\left({s}{^{\prime}}+{u}{^{\prime}}\right)\left({r}{^{\prime}}+{t}{^{\prime}}\right)\right]\left[\left(t+u\right)\left(r+s\right)+\left({t}{^{\prime}}+{u}{^{\prime}}\right)\left({r}{^{\prime}}+{s}{^{\prime}}\right)\right]},$$

where the numerator is the square of the average of the covariances for the two parental lines, while the denominator is the product of the average of the variances. Note that the constant 2^2^ in the numerator of Eq. (1) and 2^2^ in the demoninator of Eq. (1) (2 for each variance) cancelled out in the derivation of the equation.

### Genotype-based squared correlation

The following inputs are required to derive the genotype-based ${r}^{2}$ in crossbreds: genotype frequencies and the expectations of squares and cross products of genotype dosage, 0, 1, and 2, in crossbreds. Using the haplotype frequencies in Table 1 and the assumption that individuals of line $A$ mate at random to individuals of line $B$, we find the genotype frequencies in the crossbred population as shown in Table 2. Next, using these genotype frequencies, Table 3 shows the expectations of squares and cross products of genotype dosages. Computations of the expectations of combinations of genotypic values are in Appendix 1.

**Table 2:** Expected genotype frequencies in the crossbred offspring when individuals from lines ${A}$ and ${B}$ are mated at random to each other

Line ${A}$ haplotype	Line ${B}$ haplotype			
	$00\,{r}{^{\prime}}$	01 ${s}{^{\prime}}$	$10\,{t}{^{\prime}}$	$11\,{u}{^{\prime}}$
$00$ $r$^a^	$\frac{00}{00}{{r}^{\prime}r}^{\mathrm{b}}$	$\frac{00}{01}s{^{\prime}}r$	$\frac{00}{10}{t}{^{\prime}}r$	$\frac{00}{11}{u}{^{\prime}}r$
$01\,s$	$\frac{01}{00}r{^{\prime}}s$	$\frac{01}{01}s{^{\prime}}s$	$\frac{01}{10}{t}{^{\prime}}s$	$\frac{01}{11}{u}{^{\prime}}s$
$10\,t$	$\frac{10}{00}r{^{\prime}}t$	$\frac{10}{01}s{^{\prime}}t$	$\frac{10}{10}{t}{^{\prime}}t$	$\frac{10}{11}u{^{\prime}}t$
$11\,u$	$\frac{11}{00}r{^{\prime}}u$	$\frac{11}{01}s{^{\prime}}u$	$\frac{11}{10}{t}{^{\prime}}u$	$\frac{11}{11}u{^{\prime}}u$

^a^Marginal frequencies are haplotype frequencies

^b^Joint frequencies are the genotype frequencies

**Table 3:** Unordered genotypes, their genotype dosages, frequencies, and expectations of genotype dosages, and squares and cross products of genotype dosages, for locus *M* and *N*, in the crossbred offspring from random mating between lines A and B

Genotype dosage		Frequency	Expectations				
${M}_{g}$	${N}_{g}$	$f$	$E\left({M}_{g}^{2}\right)$^a^	$E\left({M}_{g}\right)$	$E\left({N}_{g}^{2}\right)$	$E\left({N}_{g}\right)$	$E\left({{M}_{g}N}_{g}\right)$
0	0	$rr{^{\prime}}$	0	0	0	0	0
0	1	$r{^{\prime}}s+rs{^{\prime}}$	0	0	${r}{^{\prime}}s+r{s}{^{\prime}}$	${r}{^{\prime}}s+r{s}{^{\prime}}$	0
0	2	$s{^{\prime}}s$	0	0	$4s{^{\prime}}s$	$2s{^{\prime}}s$	0
1	0	$r{^{\prime}}t+rt{^{\prime}}$	$r{^{\prime}}t+r{t}{^{\prime}}$	${r}{^{\prime}}t+rt{^{\prime}}$	0	0	0
1	1	${r}{^{\prime}}u+ru{^{\prime}}+{s}{^{\prime}}t+st{^{\prime}}$	$r{^{\prime}}u+ru{^{\prime}}+s{^{\prime}}t+st{^{\prime}}$	$r{^{\prime}}u+ru{^{\prime}}+s{^{\prime}}t+s{t}{^{\prime}}$	$r{^{\prime}}u+ru{^{\prime}}+{s}{^{\prime}}t+st{^{\prime}}$	${r}{^{\prime}}u+r{u}{^{\prime}}+s{^{\prime}}t+s{t}{^{\prime}}$	$r{^{\prime}}u+r{u}{^{\prime}}+s{^{\prime}}t+st{^{\prime}}$
1	2	$s{^{\prime}}u+su{^{\prime}}$	$s{^{\prime}}u+su{^{\prime}}$	$s{^{\prime}}u+s{u}{^{\prime}}$	$4s{^{\prime}}u+4su{^{\prime}}$	$2{s}{^{\prime}}u+2s{u}{^{\prime}}$	$2s{^{\prime}}u+2su{^{\prime}}$
2	0	$t{^{\prime}}t$	$4t{^{\prime}}t$	$2t{^{\prime}}t$	0	0	0
2	1	$t{^{\prime}}u+t{u}{^{\prime}}$	${4t}{^{\prime}}u+4t{u}{^{\prime}}$	$2t{^{\prime}}u+2tu{^{\prime}}$	$t{^{\prime}}u+tu{^{\prime}}$	${t}{^{\prime}}u+tu{^{\prime}}$	${2t}{^{\prime}}u+2t{u}{^{\prime}}$
2	2	$u{^{\prime}}u$	${4u}{^{\prime}}u$	${2u}{^{\prime}}u$	$4u{^{\prime}}u$	$2u{^{\prime}}u$	$4{u}{^{\prime}}u$
		1	$\left(t+u\right)+\left({t}{^{\prime}}+{u}{^{\prime}}\right)+2\left({t}{^{\prime}}+{u}{^{\prime}}\right)\left(t+u\right)$	$\left(t+u\right)+\left({t}{^{\prime}}+{u}{^{\prime}}\right)$	$\left(s+u\right)+\left({s}{^{\prime}}+u{^{\prime}}\right)+2\left({s}{^{\prime}}+{u}{^{\prime}}\right)\left(s+u\right)$	$\left(s+u\right)+\left({s}{^{\prime}}+{u}{^{\prime}}\right)$	$u{^{\prime}}\left(1+t+u\right)+u\left(1+{t}{^{\prime}}+{u}{^{\prime}}\right)+s{^{\prime}}\left(t+u\right)+s\left({t}{^{\prime}}+u{^{\prime}}\right)$ *w*

^a^$E\left({M}_{g}^{2}\right)$ refers to the expectation of the squared genotype dosage for locus$M$, and similar definitions apply for the$E\left({M}_{g}\right)$,$E\left({N}_{g}^{2}\right) ,E\left({N}_{g}\right)$,$E\left({{M}_{g}N}_{g}\right)$. The derivation of the expectations of genotype dosages is given in Appendix 2

Using the values in Table 3, the covariance of genotype dosage at the two loci follows from $cov\left({M}_{g}{N}_{g}\right)=E\left({M}_{g}{N}_{g}\right)-E\left({M}_{g}\right)E\left({N}_{g}\right)$, where ${M}_{g}$ and ${N}_{g}$ are the genotype dosages at loci $M$ and $N$, and the variances of genotype dosage follow from $var\left({M}_{g}\right)=E\left({M}_{{\varvec{g}}}^{2}\right)-{E}^{2}\left({M}_{g}\right)$ and the corresponding expression for locus $N$. Substituting the resulting expressions into the expression for the correlation coefficient yields the following expectation of the genotype-based ${r}^{2}$:

2 $${r}_{geno}^{2}=\frac{{\left[\left(u-\left(t+u\right)\left(s+u\right)\right)+\left({u}{^{\prime}}-\left({t}{^{\prime}}+{u}{^{\prime}}\right)\left({s}{^{\prime}}+{u}{^{\prime}}\right)\right)\right]}^{2}}{\left[\left(s+u\right)\left(r+t\right)+\left({s}{^{\prime}}+{u}{^{\prime}}\right)\left({r}{^{\prime}}+{t}{^{\prime}}\right)\right]\left[\left(t+u\right)\left(r+s\right)+\left({t}{^{\prime}}+{u}{^{\prime}}\right)\left({r}{^{\prime}}+{s}{^{\prime}}\right)\right]}.$$

This expression is identical to the expression for the true haplotype-based ${r}^{2}$ (Eq. (1)). Thus, when two lines (the lines can be pure or crossbred) are crossed but individuals from the two lines are mated at random to each other, expectations of the genotype-based and the haplotype-based ${r}^{2}$ in the crossbreds (F1, F2, Fn) and in other cross types (BC1, BC2, BCn) are identical, irrespective of differences in the inbreeding coefficients at the two loci. Note that our derivation also applies to other measures of LD, i.e. $D$ and $D{^{\prime}}$. For example, measures of $D$ based on genotypes and haplotypes are the numerators of Eqs. (1) and (2), which are identical. Furthermore, using Eqs. (1) and (2), the $r$ in the crossbred population can be predicted if the haplotype and genotype frequencies of the two parental lines are known.

Note that Eq. (2) refers to the expected ${r}^{2}$ between the genotype dosage at the two loci, not to an estimate thereof. Hence, although the expected values of ${r}_{geno}^{2}$ and ${r}_{hap}^{2}$ are identical, their estimates for a given data set may differ depending on sampling bias and the sampling errors of the estimates. This will be investigated using a simulation study in the next section.

### Simulation

The objective of the simulation was to investigate and compare the bias and precision of the genotype-based and haplotype-based estimates of ${r}^{2}$ for a crossbred population. We investigated the bias and the precision for different sets of allele frequencies, levels of LD as measured by ${r}^{2}$, and sample sizes. To limit computation time, we directly sampled haplotypes according to their probability distribution, rather than simulating a population of individuals. The haplotype probability distribution follows from the allele frequencies at the two loci and the level of LD. Using the haplotype frequencies and sample size, haplotypes were sampled from a multinomial distribution for each of the two parental lines. The genotypes of the crossbred individuals were obtained by random sampling of one haplotype from each line. Next, the genotype-based and haplotype-based estimates of ${r}^{2}$ were computed from the genotypes and haplotypes, respectively, of the crossbred offspring. The parameter values (allele frequencies, ${r}^{2}$ for each line, and sample size) that were used for simulation were used to compute the true ${r}^{2}$ in the crossbreds, using Eq. (1), which was used as a benchmark to evaluate the precision and bias of the two estimates of ${r}^{2}$. Thus, there were three measures of ${r}^{2}$: the true ${r}^{2}$ calculated from the parameter values used for simulation, the haplotype-based estimate of ${r}^{2}$, and the genotype-based estimate of ${r}^{2}$. For each set of parameters, results were based on 1000 replicates. We used the R software [10] to simulate the data and analyse the results. The source code for the simulation is available at the following GitHub repository. https://github.com/setegnworku/Simulation-code-for_LD_crossbred_pop.

### Scenarios investigated

We considered only biallelic loci at two loci in crossbreds resulting from the random mating of two outbred lines ($A$ and $B$). We varied three parameters: (i) allele frequencies and (ii) ${r}^{2}$ in the parental lines, and (iii) the sample size. For the allele frequencies, we considered a range from 0.05 to 0.45, incremented by 0.10, for both lines. To limit the number of scenarios, we used equal allele frequencies at the two loci for most scenarios. Note that there is no true difference between the major and the minor allele, e.g., ${p}_{A}$ = 0.05 is equivalent to ${p}_{A}$ = 0.95, such that results for allele frequencies ranging from 0.55 to 0.95 are identical to those for 0.05 to 0.45. For ${r}^{2}$ in the parental lines, we considered values of 0.2, 0.4, 0.6, and 0.8. To reduce the number of scenarios, ${r}^{2}$ was the same in both lines. We considered sample sizes of 900, 1800, and 2700. This resulted in a total of 180 scenarios with equal allele frequencies at the two loci within each line, of which 120 had different allele frequencies between the two lines, and all had equal ${r}^{2}$ in the two lines (Table 4). In addition to those 180 scenarios, we investigated a few scenarios where allele frequencies differed between loci within the parental lines and for which ${r}^{2}$ differed between the parental lines.

**Table 4:** Combinations of minor allele frequencies for lines ${A}$ and ${B}$ investigated in the simulation^a^

Line ${B}$	Line ${A}$				
	0.05	0.15	0.25	0.35	0.45
0.05	X	X	X	X	X
0.15		X	X	X	X
0.25			X	X	X
0.35				X	X
0.45					X

^a^Allele frequencies were equal for the two loci ($M$ and $N$) within a line. Apart from the diagonal elements, the allele frequencies differed between the two lines. Scenarios in this Table were replicated for sample sizes of 900, 1800, and 2700, and ${r}^{2}$ in the parental lines equal to 0.2, 0.4, 0.6, and 0.8 (equal for both lines), yielding a total of 3*4*15 = 180 scenarios

## Results and discussion

The full results for all 180 simulated scenarios, including bias, ratio of precision (ratio of standard deviation for the ${r}^{2}$ estimates using unphased genotype and haplotype data), correlation of the standard deviation, of the ${r}^{2}$ estimate using unphased genotype and haplotype data is given in the following R shiny App (https://setegnmaths.shinyapps.io/LD_App/). The source code for the Shiny App is available in the following github repository: https://github.com/setegnworku/Linkage_disequilibrium_crossbred_ShinyApp.

**Fig. 1:**
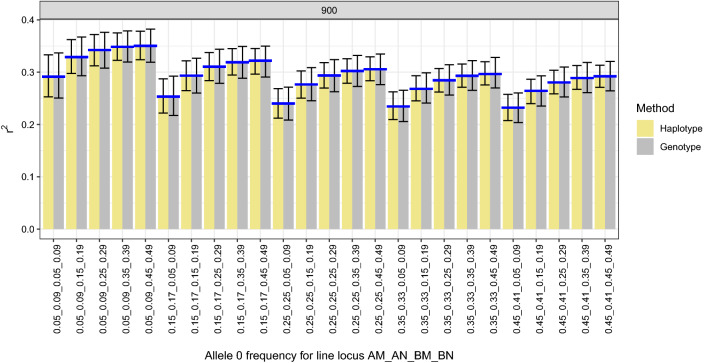
Comparison of estimates of linkage disequilibrium $({r}^{2}\pm SD)$ based on unphased genotype and haplotype data for scenarios where allele frequencies differed between loci and between lines, with ${r}^{2}=0.2$ for line $A$ and ${r}^{2}=0.4$ for line $B$. Sample size was 900 (1000 replicates)

Results showed that the estimates of ${r}^{2}$ for 180 scenarios were unbiased, both for the haplotype-based and the unphased genotype-based estimates of ${r}^{2}$. Moreover, simulation results also confirmed our theoretical finding that unphased genotype-based and haplotype-based ${r}^{2}$ on average are the same for a given dataset, irrespective of differences in inbreeding coefficients between the two loci (Fig. 1).

**Fig. 2:**
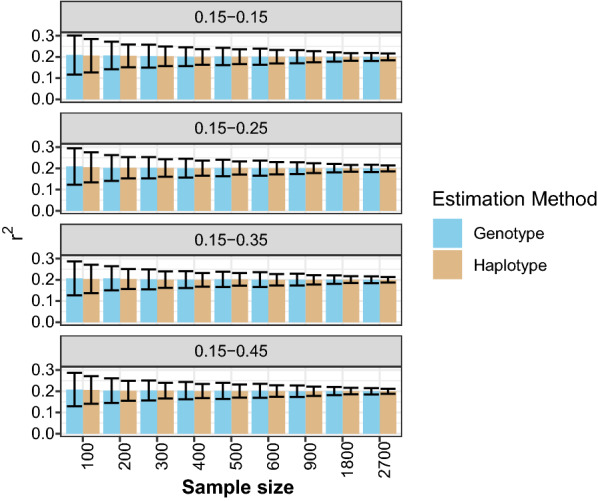
Comparison of linkage disequilibrium estimated from unphased genotype and haplotype data, for different sample sizes

As shown in Fig. 1, ${r}^{2}$ for a given dataset was unbiased for scenarios where allele frequencies differed between loci (i.e., inbreeding coefficients differed between the two loci) and between lines, and when the ${r}^{2}$ differed between the lines (0.2 and 0.4). We also tested the bias of LD estimates using unphased genotype and haplotype data for different sample sizes (Fig. 2). As shown in Fig. 2, for all scenarios, both estimators were unbiased for a sample size above 300. However, with sample size of 300 or less (100, 200, and 300), we found a small downward bias for both the unphased genotype- and haplotype-based estimates (the independent sample t-test showed the bias was significant for some of the scenarios for both the unphases genotype- and haplotype-based estimates). It is well known that the estimator of the correlation coefficient is known to be biased, and more so for smaller samples [11], which may explain the bias we found in small samples.

### Bias

For all scenarios (180), the estimates of the ${r}^{2}$ using unphased genotype and haplotype data were both unbiased. We ran an independent sample t-test to test the bias of the estimates of ${r}^{2}$ using unphased genotype and haplotype data from the true ${r}^{2}$. For all 180 scenarios, the bias of the estimates was not significantly different from zero for both methods (p value > 0.05). The average absolute bias across 180 scenarios was 0.0004 when using unphased genotype data, and 0.0003 when using haplotype data (Table 5). The maximum absolute bias across the 180 scenarios was 0.003 when using unphased genotype data and 0.002 when using haplotype data. As expected, the bias decreased as sample size increased. For example, with unphased genotype data, the average absolute bias was 0.0005 for a sample size of 900 and 0.0001 for a sample size of 2700. Corresponding values for haplotype data were 0.0003 and 0.0001. These results show that the estimators of ${r}^{2}$ are consistent for both unphased genotype data and haplotype data, because the bias of the ${r}^{2}$ estimates decreased as sample size increased.

**Table 5:** Summary of estimates of bias and precision (standard deviation) of ${r}^{2}$ using unphased genotype and haplotype data

Parameter	Haplotype	Genotype
Average absolute bias across 180 scenarios	0.0003	0.0004
Maximum absolute bias	0.002	0.003
Average absolute bias sample size 900	0.0003	0.0005
Average absolute bias sample size 2700	0.0001	0.0001
Standard deviation (SD) across 180 scenarios	0.021	0.023
Maximum SD	0.055	0.057
Average SD with sample size of 900	0.027	0.031
Average SD with sample size of 2700	0.016	0.018

### Precision

For all scenarios, estimates of LD based on haplotype data were more precise than estimates based on unphased genotype data, although the differences were small. For example, the mean standard deviation of the estimates of ${r}^{2}$ across all scenarios was 0.023 when using unphased genotype data and 0.021 when using haplotype data. The maximum standard deviation for estimates of ${r}^{2}$ across all scenarios was 0.057 using unphased genotype data and 0.055 using haplotype data. The precision of the estimates of ${r}^{2}$ increased as sample size increased, both with unphased genotype and with haplotype data. For example, the average standard deviation across all scenarios with a sample size of 900 was 0.031 with unphased genotype data and 0.027 with haplotype data. The corresponding values for a sample size of 2700 were 0.018 and 0.016. This result was as expected because the standard error of the estimate of a correlation coefficient decreases as sample size increases [12]. Thus, with a sufficient sample size, ${r}^{2}$ in crossbreds can be estimated accurately based on unphased genotype data.

**Fig. 3:**
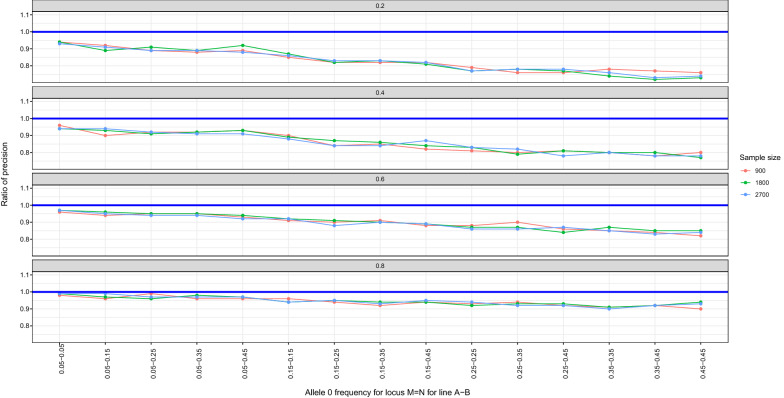
Ratio of precision for all scenarios investigated for ${r}^{2}$ values of 0.2, 0.4, 0.6, and 0.8

We further investigated in which scenarios the difference in precision for the estimates of ${r}^{2}$ using unphased genotype versus haplotype data was the largest. We investigated this by computing the ratio of the standard deviations of the estimates of ${r}^{2}$ using haplotype data and unphased genotype data. Thus, smaller values of this ratio indicate a greater superiority of estimates based on haplotypes. As shown in Fig. 3, the ratio of precision was less than 1 for all scenarios, indicating that the estimate based on haplotype data was more precise than that based on unphased genotype data. The ratio of the precision increased as the level of LD increased. For example, for an ${r}^{2}$ of 0.2, the ratio of precision ranged from 0.75 to 0.9, while with an ${r}^{2}$ of 0.8, the ratio ranged from 0.92 to 0.98. The difference between the estimates of ${r}^{2}$ based on unphased genotype vs. haplotype data originates solely from the double heterozygotes (00/11 for coupling phase, or 01/10 for repulsion phase). As ${r}^{2}$ increases, the frequencies of the coupling phase haplotypes 00 and 11 or of the repulsion phase haplotypes 01 and 10, increase, which reduces the opportunity for the haplotype method to provide extra information by distinguishing between them. As a result, at larger ${r}^{2}$, the precision of the estimates of ${r}^{2}$ using unphased genotype and haplotype data are expected to be closer to each other. On the other hand, at low ${r}^{2}$, all haplotypes (00, 01, 10, 11) are possible and the haplotype-based method provides additional information. For this reason, the estimate of ${r}^{2}$ based on haplotype data is more precise than the estimate based on unphased genotype data, in particular when the true ${r}^{2}$ is small.

**Fig. 4:**
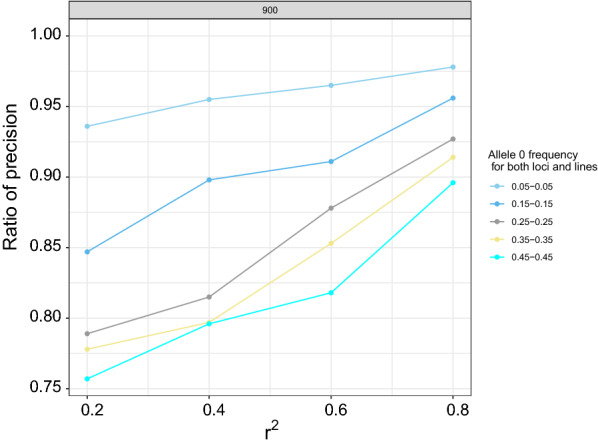
Ratio of precision for sample size of 900 for selected scenarios

The ratio of precision decreased when the minor allele frequencies for the two loci increased (Figs. 3 and 4). For example, for allele frequencies of 0.05 and 0.05 at the two loci, the ratio of precision ranged from 0.93 to 0.99, while it ranged from 0.73 to 0.94 for allele frequencies of 0.45 and 0.45. This is because the proportion of the double heterozygotes in the population decreases when the minor allele frequencies at the two loci decrease, which reduces the extra information provided by the haplotype-based method**.** This is in agreement with [13]. There was also an interaction between the level of LD and the minor allele frequency, with the ratio of precision increasing when the level of LD increased but this increase was larger for higher values of the minor allele frequency (Fig. 4). The ratio of precision at allele frequencies of 0.05 and 0.05 was 0.91 when ${r}^{2}$ was 0.2 and 0.99 for an ${r}^{2}$ of 0.9. However, the corresponding values for allele frequencies of 0.45 and 0.45 were 0.70 when ${r}^{2}$ was 0.2 and 0.94 for an ${r}^{2}$ of 0.9. When the minor allele frequencies at the two loci decrease, the proportion of double heterozygotes decreases, which reduces the extra information provided by the haplotype-based method. Thus, with extreme allele frequencies at the loci (e.g. 0.05 and 0.05), both methods yielded similar results, irrespective of the level of LD. On the other hand, at intermediate allele frequencies, such as 0.45 and 0.45, the proportion of double heterozygotes in the population increases, which increases the extra information provided by the haplotype-based method, particularly when LD is weak.

In real applications, the true ${r}^{2}$ is unknown and the ${r}^{2}$ computed using haplotype data would serve as the reference value. In that case, the comparison would be between the ${r}^{2}$ computed using unphased genotype data relative to the estimate based on haplotype data. In this case, the average absolute bias across the 180 scenarios using unphased genotype was very close to zero (0.00017) and the average standard deviation of estimates based on unphased genotype data across all scenarios relative to haplotype data was 0.0026. In addition, the haplotype-based method assumes that the haplotype can be determined without error for each individual, which means that in reality the absolute bias may be lower than the above value of 0.00017, depending on the error of haplotype estimation. Thus, estimates of ${r}^{2}$ computed using unphased genotype and haplotype data are indistinguishable in terms of both bias and precision in practice, particularly with sufficient sample size.

This paper extends the work of Rogers and Huff [8] and Weir [14], who showed that LD can be estimated from unphased genotype data when the allele frequency in line $A$ and line $B$ is the same, and when the inbreeding coefficient is identical for the two loci. Here, we showed that LD can also be estimated using unphased genotype data when the allele frequencies differ between lines $A$ and $B$ and the inbreeding coefficients differ between the two loci. This is particularly relevant for hybrids in plant breeding [15] and for crossbreds in animal breeding [16, 17].

## Conclusions

This work shows that the expectation of estimates of linkage disequilibrum (LD) between loci based on unphased genotypes and haplotypes in F1 crossbreds are identical. Estimates of LD, i.e. ${r}^{2}$, are more precise and less biased when based on haplotype data compared to unphased genotype data. For both unphased genotype and haplotype data, the precision of ${r}^{2}$ increases and the bias of the estimates decreases as sample size increases. More importantly, the difference in precision and bias between estimates of ${r}^{2}$ using haplotype and unphased genotype data decreases as sample size increases. Thus, LD in a crossbred population can be estimated using unphased genotyped data with little bias and good precision, particularly with sufficient sample size.

## Appendix 1

This appendix shows under which conditions the inbreeding coefficients at the two loci ($M$ and $N$) are equal when two outbred lines ($A$ and $B$) mate randomly. The alleles are denoted *0* and *1*, ${p}_{AM}$ is the frequency of allele *1* at locus $M$ in line $A$, and ${p}_{BM}$ is the frequency of allele *1* at locus $M$ in line $B$.

To simplify the symbols notation let $a={p}_{AM}$, $b={p}_{BM}$, $c={p}_{AN}$, and $d={p}_{BN}$. The inbreeding coefficient for locus $M$ is ${f}_{M}=\frac{-{\left(\mathrm{a}-\mathrm{b}\right)}^{2}}{\left(a+b\right)\left(2-a+b\right)}$ and for locus $N$ is ${f}_{N}=\frac{-{\left(c-d\right)}^{2}}{\left(\mathrm{c}+\mathrm{d}\right)\left(2-c+d\right)}$. By solving the expression ${f}_{N}={f}_{M}$, using Wolfram Mathematica (www.wolfram.com), we get the following complex solutions:

$$\left\{d\to \frac{{a}^{2}-2ab+{b}^{2}+2ac-2{a}^{2}c+2bc-2{b}^{2}c-\left(a-b\right)\sqrt{{a}^{2}-2ab+{b}^{2}+8ac-4{a}^{2}c+8bc-8abc-4{b}^{2}c-8a{c}^{2}+4{a}^{2}{c}^{2}-8b{c}^{2}+8ab{c}^{2}+4{b}^{2}{c}^{2}}}{2\left(a+b-2ab\right)}\right\},$$

$$\left\{ {a \to \frac{b}{{ - 1 + 2b}},d \to \frac{c}{{ - 1 + 2c}}} \right\},\left. {\left\{ {a \to 0,b \to 0} \right\},\left\{ {a \to 1,b \to 1} \right\}} \right.$$

Conclusion: there are two trivial solutions, i.e. p = 0($(\left\{a\to 0,b\to 0\right\}),$ and p = 1$(\left\{a\to 1,b\to 1\right\})$; two simple solutions $(\left\{a\to \frac{b}{-1+2b},d\to \frac{c}{-1+2c}\right\},)$, and two rather complex solutions; thus, in general the two inbreeding coefficients are different.

## Appendix 2

This appendix shows the computation of the expectation for different linear combination of genotypic values ${M}_{g}$ and ${N}_{g}$ at loci $M$ and $N$, respectively, as indicated in Table 3.

### Computation of ${\varvec{E}}\left({{\varvec{M}}}_{{\varvec{g}}}\right)$

$$E\left({M}_{g}\right)={r}{^{\prime}}t+{rt}{^{\prime}}+{r}{^{\prime}}u+r{u}{^{\prime}}+{s}{^{\prime}}t+s{t}{^{\prime}}+{s}{^{\prime}}u+s{u}{^{\prime}}+{2t}{^{\prime}}t+{2u}{^{\prime}}t+2u{t}{^{\prime}}+{2u}{^{\prime}}u$$

$$E\left({M}_{g}\right)={r}{^{\prime}}\left(t+u\right)+{t}{^{\prime}}\left(r+s\right)+{u}{^{\prime}}\left(r+s\right)+{s}{^{\prime}}\left(t+u\right)+2t\left({t}{^{\prime}}+{u}{^{\prime}}\right)+2u\left({t}{^{\prime}}+{u}{^{\prime}}\right)$$

$$E\left({M}_{g}\right)=\left({r}^{\prime}+s^{\prime}\right)\left(t+u\right)+\left({t}^{\prime}+u^{\prime}\right)\left(r+s\right)+2\left(t+u\right)\left({t}^{\prime}+u^{\prime}\right).$$

Simplifying this yields:

3 $$E\left({M}_{g}\right)=\left(t+u\right)+\left({t}{^{\prime}}+u{^{\prime}}\right).$$

### Computation of ${\varvec{E}}\left({{\varvec{M}}}_{{\varvec{g}}}^{2}\right)$

$$E\left({M}_{g}^{2}\right)={r}{^{\prime}}t+{rt}{^{\prime}}+{r}{^{\prime}}u+r{u}{^{\prime}}+{s}{^{\prime}}t+s{t}{^{\prime}}+{s}{^{\prime}}u+s{u}{^{\prime}}+{4t}{^{\prime}}t+{4u}{^{\prime}}t+4u{t}{^{\prime}}+{4u}{^{\prime}}u$$

$$E\left({M}_{g}^{2}\right)={r}{^{\prime}}\left(t+u\right)+{t}{^{\prime}}\left(r+s\right)+{u}{^{\prime}}\left(r+s\right)+{s}{^{\prime}}\left(u+t\right)+4{t}{^{\prime}}\left(t+u\right)+4{u}{^{\prime}}\left(t+u\right)$$

$$E\left({M}_{g}^{2}\right)={r}^{\prime}\left(t+u\right)+{t}^{\prime}\left(r+s\right)+{u}^{\prime}\left(r+s\right)+{s}^{\prime}\left(u+t\right)+2\left({t}^{\prime}+{u}^{\prime}\right)\left(t+u\right)+2\left({t}^{\prime}+{u}^{\prime}\right)\left(t+u\right).$$

Simplifying this yields:

4 $$E\left({M}_{g}^{2}\right)=\left(t+u\right)+\left({t}{^{\prime}}+{u}{^{\prime}}\right)+2\left(t+u\right)\left({t}{^{\prime}}+{u}{^{\prime}}\right).$$

### Computation of ${\varvec{E}}\left({{\varvec{N}}}_{{\varvec{g}}}\right)$

$$E\left({N}_{g}\right)={r}{^{\prime}}s+{rs}{^{\prime}}+2{s}{^{\prime}}s+{r}{^{\prime}}u+r{u}{^{\prime}}+{s}{^{\prime}}t+s{t}{^{\prime}}+2{s}{^{\prime}}u+2s{u}{^{\prime}}+t{u}{^{\prime}}+{t}{^{\prime}}u+{2u}{^{\prime}}u$$

$$E\left({N}_{g}\right)={r}{^{\prime}}\left(s+u\right)+{s}{^{\prime}}\left(r+t\right)+{u}{^{\prime}}\left(r+t\right)+{t}{^{\prime}}\left(s+u\right)+2{s}{^{\prime}}u+2{s}{^{\prime}}s+2{su}{^{\prime}}+2u{^{\prime}}u$$

$$E\left({N}_{g}\right)=\left({r}{^{\prime}}+{t}{^{\prime}}\right)\left(s+u\right)+\left({s}{^{\prime}}+{u}{^{\prime}}\right)\left(r+t\right)+2\left({s}{^{\prime}}+{u}{^{\prime}}\right)\left(s+u\right)$$

$$E\left({N}_{g}\right)=\left({r}^{\prime}+{t}^{\prime}\right)\left(s+u\right)+\left({s}^{\prime}+{u}^{\prime}\right)\left(r+t\right)+\left({s}^{\prime}+{u}^{\prime}\right)\left(s+u\right)+\left({s}^{\prime}+{u}^{\prime}\right)\left(s+u\right).$$

Simplifying this yields

5 $$E\left({N}_{g}\right)=\left(s+u\right)+\left({s}{^{\prime}}+{u}{^{\prime}}\right).$$

### Computation of ${\varvec{E}}\left({{\varvec{N}}}_{{\varvec{g}}}^{2}\right)$

$$E\left({N}_{g}^{2}\right)={r}{^{\prime}}s+{rs}{^{\prime}}+{r}{^{\prime}}u+r{u}{^{\prime}}+{s}{^{\prime}}t+s{t}{^{\prime}}+{t}{^{\prime}}u+t{u}{^{\prime}}+4{s}{^{\prime}}s+4{s}{^{\prime}}u+4s{u}{^{\prime}}+{4u}{^{\prime}}u$$

$$E\left({N}_{g}^{2}\right)={r}{^{\prime}}s+{r}{^{\prime}}u+{rs}{^{\prime}}+{s}{^{\prime}}t+r{u}{^{\prime}}+s{t}{^{\prime}}+{t}{^{\prime}}u+t{u}{^{\prime}}+4{s}{^{\prime}}\left(s+u\right)+4{u}{^{\prime}}\left(s+u\right)$$

$$E\left({N}_{g}^{2}\right)={r}{^{\prime}}\left(s+u\right)+{s}{^{\prime}}\left(r+t\right)+{u}{^{\prime}}\left(r+t\right)+{t}{^{\prime}}\left(s+u\right)+4\left(s+u\right)\left({s}{^{\prime}}+{u}{^{\prime}}\right)$$

$$E\left({N}_{g}^{2}\right)={r}^{\prime}\left(s+u\right)+{s}^{\prime}\left(r+t\right)+{u}^{\prime}\left(r+t\right)+{t}^{\prime}\left(s+u\right)+2\left(s+u\right)\left({s}^{\prime}+{u}^{\prime}\right)+2\left(s+u\right)\left({s}^{\prime}+{u}^{\prime}\right).$$

Simplifying this yields:

6 $$E\left({N}_{g}^{2}\right)=\left(s+u\right)+\left({s}{^{\prime}}+{u}{^{\prime}}\right)+2\left(s+u\right)+\left({s}{^{\prime}}+{u}{^{\prime}}\right).$$

### Computation of ${\varvec{E}}\left({{{\varvec{M}}}_{{\varvec{g}}}{\varvec{N}}}_{{\varvec{g}}}\right)$

$$E\left({{M}_{g}{N}_{g}}\right)={r}{^{\prime}}u+{ru}{^{\prime}}+{t}{^{\prime}}s+t{s}{^{\prime}}+{2s}{^{\prime}}u+2s{u}{^{\prime}}+{2t}{^{\prime}}u+2{u}{^{\prime}}t+{4u}{^{\prime}}u$$

$$E\left({{M}_{g}{N}_{g}}\right)=u\left({r}^{\prime}+{s}^{\prime}\right)+2u\left({t}^{\prime}+{u}^{\prime}\right)+{u}^{\prime}\left(r+s\right)+2{u}^{\prime}\left(t+u\right)+s\left({t}^{\prime}+{u}^{\prime}\right)+{s}^{\prime}\left(t+u\right).$$

Simplifying this yields:

7 $$E\left({{M}_{g}{N}_{g}}\right)=u\left(1+{t}{^{\prime}}+{u}{^{\prime}}\right)+{u}{^{\prime}}\left(1+t+u\right)+s\left({t}{^{\prime}}+{u}{^{\prime}}\right)+{s}{^{\prime}}\left(t+u\right).$$
